# Bergaptol, a Major Furocoumarin in Citrus: Pharmacological Properties and Toxicity

**DOI:** 10.3390/molecules29030713

**Published:** 2024-02-04

**Authors:** Pakkapong Phucharoenrak, Dunyaporn Trachootham

**Affiliations:** Institute of Nutrition, Mahidol University, Nakhon Pathom 73170, Thailand; pakkapong.phu@mahidol.ac.th

**Keywords:** bergaptol, furanocoumarin, citrus, pharmacologic actions, toxicity

## Abstract

Bergaptol (5-hydroxypsoralen or 5-hydroxyfuranocoumarin) is a naturally occurring furanocoumarin widely found in citrus fruits, which has multiple health benefits. Nonetheless, no specific review articles on bergaptol have been published. Compiling updated information on bergaptol is crucial in guiding future research direction and application. The present review focuses on the research evidence related to the pharmacological properties and toxicity of bergaptol. Bergaptol has anti-inflammatory, antioxidant, anti-cancer, anti-osteoporosis, anti-microbial, and anti-lipidemic effects. It can inhibit the activities of cytochrome P450s (CYP), especially CYP2C9 and CYP3A4, thereby affecting the metabolism and concentrations of some drugs and toxins. Compared with other coumarins, bergaptol has the least potency to inhibit CYP3A4 in cancer cells. Instead, it can suppress drug efflux transporters, such as P-glycoprotein, thereby overcoming chemotherapeutic drug resistance. Furthermore, bergaptol has antimicrobial effects with a high potential for inhibition of quorum sensing. In vivo, bergaptol can be retained in plasma for longer than other coumarins. Nevertheless, its toxicity has not been clearly reported. In vitro study suggests that, unlike most furocoumarins, bergaptol is not phototoxic or photomutagenic. Existing research on bergaptol has mostly been conducted in vitro. Further in vivo and clinical studies are warranted to identify the safe and effective doses of bergaptol for its multimodal application.

## 1. Introduction

**Figure 1:**
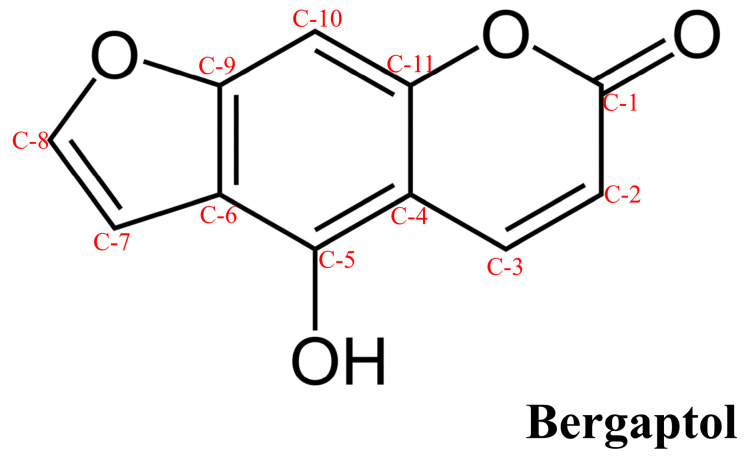
Chemical structure of bergaptol.

Bergaptol (CAS No: 486-60-2, PubChem CID: 5280371), also known as 5-hydroxypsoralen or 5-hydroxyfuranocoumarin, belongs to the class of furanocoumarins. It is a primary essential metabolite that plays an important role in the growth, development, and reproduction of plants. The molecular formula of bergaptol is C_11_H_6_O_4_ and its molecular weight is 202.16 g/mol. Its structure is composed of psoralens that contain a hydroxyl group attached at its C-5 position (Figure 1).

**Figure 2:**
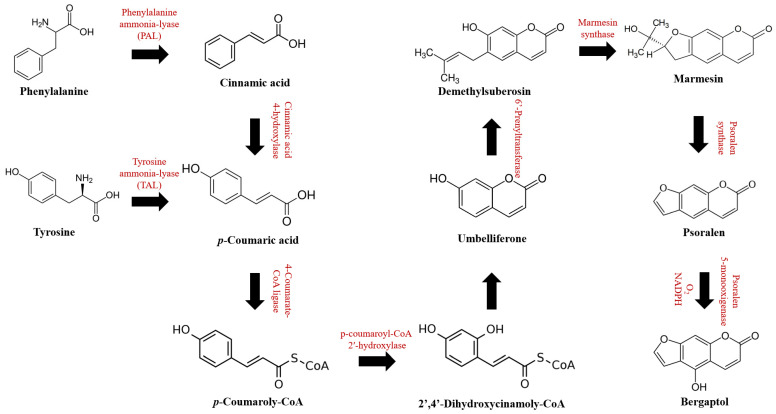
Biosynthetic pathways of bergaptol.

The biosynthesis pathway of bergaptol (shown in Figure 2) starts from the formation of p-coumaric acid, which can either be converted from cinnamic acid catalyzed by the enzyme cinnamic acid 4-hydroxylase or from tyrosine catalyzed by the enzyme tyrosine amino lyase (TAL). Cinnamic acid is derived from phenylalanine catalyzed by the enzyme phenylalanine ammonia-lyase (PAL). The p-Coumaric acid is further catalyzed through thioesterification by 4-coumarate-CoA ligase, then ortho-hydroxylation by cinnamoyl-CoA 2′-hydroxylase for the formation of n 2′, 4′-Dihydroxycinamoly-CoA. The ortho-hydroxylation is a key step in coumarin biosynthesis [1,2]. It occurs by parahydroxylation of the phenylpropanoid precursor (e.g., cinnamic acid) [3]. The by-product 2′,4′-Dihydroxycinamoly-CoA can be spontaneously converted further to umbelliferone by the closure of the lactone ring [4]. Umbelliferone is the precursor of various coumarin derivatives, including bergaptol. The formation starts with sequential conversion to demethylsuberosin, then it is further transformed to marmesin and psoralen by two consecutive cytochrome P450 monooxygenases (marmesin synthase and psoralen synthase) [5]. Finally, the hydroxyl group is added to the C-5 position of psoralen to form bergaptol. For chemical synthesis, bergaptol can be synthesized from bergapten. First, bergapten can be generated from visnagin, 4-Methoxy-6-hydroxybenzofuran-5-carboxaldehyde, or phloroglucinol [6,7]. Then, the synthesized bergapten can be further derivatized with boron tribromide (BBr_3_) in dichloromethane or degraded with heat to yield bergaptol [8,9].

Bergaptol has been detected in several different foods including cereals, teas, mushrooms, fruits, vegetables, and aquatic products [10]. The major natural sources of bergaptol are citrus plants (*Citrus* spp.). Bergaptol is most abundant in essential oils from citrus peel, such as sweet orange (*Citrus sinensis*) [11], bergamot (*Citrus bergamia*) [12], lemon (*Citrus limon*) [13], and lime (*Citrus aurantiifolia*) [14]. However, in grapefruit (*Citrus paradisi*), it is mostly found in juice [15]. Interestingly, some fungi, such as *Penicillium ulaiense*, *Lasiodiplodia theobromae*, and *Geotrichum candidum*, can also synthesize bergaptol [16].

At present, furanocoumarins (e.g., bergaptol, bergapten, bergamottin, etc.) are gaining more and more attention from researchers. However, descriptions of the health benefits and adverse effects of bergaptol have not been compiled together. This review aims to provide a comprehensive summary and discussion of the pharmacological properties and toxicities of bergaptol. Future research directions are proposed.

## 2. Chemical Properties, Bioavailability, and Pharmacokinetics

Bergaptol is stable in solid form. It is soluble in organic solvents such as dimethyl sulfoxide (DMSO) at approximately 40 mg/ mL and in dimethylformamide at 20 mg/ mL [17,18,19]. It is sparingly soluble in ethanol at approximately 2 mg/mL and practically insoluble in water (solubility at less than 1 mg/mL) [10,17]. The exact melting point and boiling point can vary based on several factors including specific forms and pressure. Generally, its melting point is around 287 ± 10 °C. Interestingly, bergaptol decomposes before reaching boiling point. As a result, there is no apparent boiling point under normal atmospheric pressure (760 mmHg). Predicted data from ACD/Labs Percepta Platform-PhysChem Module indicated that the boiling point of bergaptol is around 311.9 ± 11.0 °C at 760 mmHg [18]. Since it can be decomposed when heated below its boiling point, a suitable method for extraction of such compounds is vacuum distillation, in which the pressure is decreased to less than its vapor pressure, causing evaporation of most volatile liquids [20].

Using the Caco-2 cell model of intestinal absorption, Yang et al. found that bergaptol in *Angelicae pubescentis* radix extract had bidirectional apparent permeability coefficients (*P*app) of 16.87 ± 1.64 × 10^−6^ cm/s for apical to basolateral, and 19.96 ± 1.55 × 10^−6^ cm/s for basolateral to apical [21]. Since the *P*app values were more than 10 × 10^−6^ cm/s, bergaptol was considered well-absorbed [21]. Nevertheless, a pharmacokinetic study in rats showed that bergaptol exhibited a longer half-life compared with other coumarin compounds (7.10 ± 1.83 h) and reached its maximum concentration of 25.40 ± 8.86 μg/L at 4.40 ± 0.89 h. The apparent volume of distribution and clearance rates were 14.30 ± 4.93 L/Kg and 1.44 ± 0.54 L/h/Kg, respectively [21]. Such kinetic parameters suggest that bergaptol is moderately absorbed and slowly cleared, so it can be retained in plasma for longer than the other 15 types of coumarin. In humans, bergaptol was measurable in plasma starting from 15 min and retained for up to 3 h [22]. Furthermore, an in vitro study demonstrated that the fungus *Aspergillus niger* can metabolize bergaptol into a water-soluble form (bergaptol-5-sulfate) through sulfate conjugation [23]. This finding aligns with clinical kinetic studies in humans that reported renal excretion of bergaptol in both free and conjugated forms in the urine [22,24]. A study found that after consuming grapefruit and grapefruit juice, bergaptol and its metabolites (sulfate and glucuronide conjugate) were detected in urine as early as 1 h, reached a peak at 3–5 h, and remained in urine in small concentrations as late as 24 h after ingestion [22]. Another study found that within 6 h after consuming 900 mL commercial grapefruit juice (containing 12.5 mg bergaptol), healthy volunteers excreted 0.36 mg free bergaptol and 13.23 mg conjugated bergaptol [22].

## 3. Pharmacological Properties

### 3.1. Anti-Inflammatory Effects

**Figure 3:**
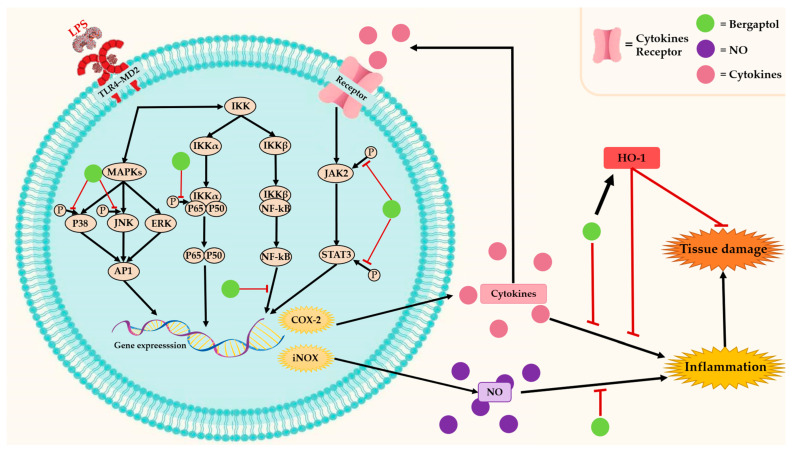
The anti-inflammatory mechanisms of bergaptol. 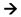 indicates activation, 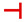 indicates inhibition.

Inflammation is a biological defense mechanism in response to a variety of harmful stimuli such as pathogens, microorganisms, damaged cells, trauma, toxic compounds, or metabolic stress [25]. Nevertheless, uncontrolled inflammation may contribute to many chronic inflammatory diseases such as cardiovascular disease, chronic obstructive pulmonary disease (COPD), rheumatoid arthritis, allergies, cancer, etc. [26,27]. Traditional herbal medicines that contain bergaptol have anti-inflammatory properties [28,29,30]. Several pieces of evidence suggest that bergaptol may interfere with cyclooxygenase-2 (COX-2), heme oxygenase 1 (HO-1), and other inflammatory signaling pathways (Figure 3) [30,31]. These enzymes play a crucial role in inflammatory events such as the synthesis of inflammatory mediators, production of inflammatory cytokines, leukocyte adhesion and migration, etc. [32,33]. Thus, inhibition of these enzymes by bergaptol can potentially reduce inflammation.

Bergaptol isolated from blossoms of bitter orange *(Citrus aurantium* L. var. *amara* Engl.) significantly decreased the levels of tumor necrosis factor-α (TNF-α) and interleukin (IL-1β and IL-6) and inhibited the expression of inflammatory factors in lipopolysaccharide (LPS)-induced RAW264.7 mouse macrophages [31]. Furthermore, bergaptol can inhibit NO production and its accumulation by suppressing inducible nitric oxide synthase (*iNOS*) gene expression. The inhibitory mechanism involves blocking the phosphorylation levels of c-Jun N-terminal kinase (JNK) and p38 of the mitogen-activated protein kinase (MAPK) family and suppressing the activation of nuclear factor kappa-light-chain-enhancer of activated B cells (NF-κB) [31,34]. Recently, a study in mice models of LPS-induced neuronal damage showed that bergaptol can reduce neurological damage and improve cognitive impairment [35]. The mechanism involves the prevention of dendritic spine reduction and suppression of inflammatory factor production in the hippocampus [35]. These effects are attributed to the downregulation of *TNF-α*, *IL*-6, and *IL-1β* mRNA levels, as well as the reduction in phosphorylation levels of JAK2, STAT3, and p65 [35]. Although the potential anti-inflammatory effects of bergaptol have received increasing attention from researchers, currently available data for a comprehensive understanding are still limited. Therefore, further research is needed to thoroughly elucidate the mechanism of action, which could lead to therapeutic applications.

### 3.2. Antioxidative Effects

Oxidative stress is an imbalance between production and accumulation of reactive oxygen species (ROS). It can lead to damage in several physiological conditions [36]. A previous study suggested that bergaptol had a strong antioxidant activity with superior free-radical-scavenging activity than that of Trolox, the water-soluble analog of vitamin E [37]. It can inactivate the hydroxyl radical (·OH) through multiple mechanisms, including hydrogen atom transfer (HAT), single-electron transfer (SET), sequential proton loss electron transfer (SPLET), and radical adduct formation (RAF) mechanisms [37,38]. Previous studies found that the antioxidant mechanism depends on the polarity of the medium, which affects the polarity of the antioxidant compound [39,40]. In the case of bergaptol, the thermodynamic parameters suggested that HAT was the major reaction mechanism in a nonpolar medium (e.g., benzene), while SPLET was the major reaction mechanism in a polar medium (e.g., water) [40]. In addition, bergaptol reacts with and reduces several types of ROS including hydroxyl radical (·OH), methoxy radical (·OCH_3_), isopropoxy radical (·OCH(CH_3_)_2_), hydroperoxyl radical (·OOH), vinyl peroxy radical (·OOCH=CH_2_), and methyl peroxy radical (·OOCH_3_) [40]. Furthermore, bergaptol has been shown to not only inhibit various types of free radicals but also reduce the oxidation of low-density lipoprotein (LDL) [31]. Scientific evidence at the in vivo and clinical levels indicates that preventing LDL oxidation or increasing the resistance of oxidized LDL is one of the ways to mitigate the risk of atherosclerosis [41]. Many bergaptol-containing plant extracts (e.g., *Ficus infectoria*, *Citrus paradise*, *Citrus sinensis*, *Citrus bergamia*, and *Citrus limon*) have potent antioxidant activities, according to studies using several assays [42,43,44,45]. Moreover, bergaptol-rich lemon essential oil was shown to enhance the activities of several antioxidant enzymes in mice, such as superoxide dismutase (SOD) and phospholipid hydroperoxide glutathione peroxidase (GSH-Px), through the upregulation of mRNA and protein expressions [45]. This led to an increase in serum total antioxidant capacity in mice. Though existing literature suggests an impressive in vitro antioxidant effect of bergaptol and bergaptol-containing fruits, the clinical antioxidative capacity of bergaptol has not been studied. Owing to its powerful antioxidant effects, further exploring the application of bergaptol in ROS-stress-related diseases is worthwhile.

### 3.3. Anti-Cancer Effects

**Figure 4:**
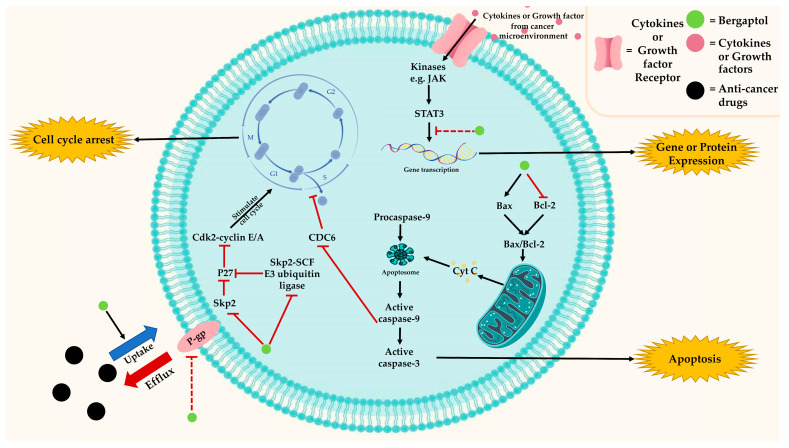
The anti-cancer properties mechanisms of bergaptol. 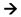 indicates activation, 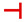 indicates inhibition, and 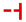 indicates possibility of inhibition.

Cancer is one of the most life-threatening diseases and a major cause of death globally. In 2020, 18 million new cases and 9.9 million deaths were reported worldwide [46]. Several natural compounds have shown promising results in their anti-cancer properties and have gained attention for development in cancer therapy [47]. Bergaptol is one of the natural compounds that has been reported to possess anti-cancer properties through various mechanisms that lead to apoptosis induction or cell cycle arrest. Figure 4 summarizes the anti-cancer effects of bergaptol, which include blocking transcription factors, e.g., STAT3, modulating apoptotic regulators, e.g., BAX/BCL2, stabilizing cell-cycle inhibitors, e.g., p27, and blocking p-glycoprotein-mediated drug efflux.

In vitro cytotoxicity assays showed that bergaptol can suppress proliferation of several types of cancer cells with half maximal inhibitory concentration (IC_50_) between 10–60 μM; e.g., U87 human glioblastoma cells (IC_50_ = 10.67 μM), A549 human lung carcinoma cells (IC_50_ = 26.42 μM), HeLa human adenocarcinoma cells (IC_50_ = 58.57 μM), Hep G2 human hepatocellular carcinoma cells (IC_50_ = 68.42 μM), and MCF-7 human breast cancer cells (IC_50_ = 52.2 μM) [48,49,50]. Our recent in vitro study showed that ethanolic extract of lime peel can induce apoptosis of hotspot p53 mutated hepatocellular carcinoma cells, PLC/PRF5. Interestingly, the mechanisms probably involved synergistic effects between multiple bioactive compounds. Among those compounds, a very high amount of bergaptol was present in the extract [47].

In MCF-7 breast cancer cells, bergaptol can induce apoptosis via the mitochondrial cell death pathway [49]. It has been shown to induce cell apoptosis by increasing the gene expression of pro-apoptotic protein *Bax* and decreasing the gene expression of anti-apoptotic protein *Bcl*-2 expression in a dose-dependent relationship [49]. Such a reduction in the transcription rate of Bax/Bcl-2 results in cytochrome c release from mitochondria to cytosol and consequently activation of caspase-9 and caspase-3 [49]. The activated caspase-3 not only induces apoptosis but also causes cell cycle arrest by cleavage of cell division cycle 6 (CDC6), an essential regulator of DNA replication at the G1/S phase [49,51]. Moreover, bergaptol could inhibit the activity of Skp2-SCF E3 ubiquitin ligase and decrease the level of Skp2 protein, leading to reduced protein degradation and consequent stabilization of cyclin-dependent kinase inhibitor p27, a key regulator of the cell cycle [52,53]. This potential is supported by the study of ethanolic extract of pomelo (*Citrus grandis Tomentosa*) in A549 human lung carcinoma cells and an LLC tumor mouse model [52]. These results also suggest that bergaptol may influence changes in various molecular pathways contributing to the inhibition of cell proliferation by promoting G1 phase cell cycle arrest.

Signal transducer and activator of transcription 3 (STAT3) is a complex multifunctional transcription factor that affects cellular metabolism and immune responses [54]. STAT3 signaling participates in cancer promotion by regulating glycolysis, oxidative phosphorylation, ROS production, and lipid and glutamine metabolisms in both cancer cells and other cells in the tumor microenvironment [55]. Alteration of cellular metabolism of immune cells could lead to increased cancer migration and metastasis [54,56]. Recent studies have shown that furanocoumarins, including bergaptol, bergapten, and bergamottin, display potent activity in inhibiting STAT3 protein expression [15,35,57].

Owing to the several hallmarks and redundant pathways in cancer cells, simultaneous targeting of multiple pathways is required to achieve a favorable outcome for cancer patients [58]. Therefore, combinatorial chemotherapy is the cornerstone of cancer treatment, which not only reduces adverse effects but may also help overcome drug resistance [58,59]. A recent study showed that 10 μM of bergaptol caused up to 270% enhancement in the cellular uptake of a chemotherapeutic drug, vinblastine, in Caco-2 cells [60]. Vinblastine is a natural vinca alkaloid used in systemic treatments for a variety of cancers [61]. Interestingly, compared with FC726, DHBG, bergamottin, and bergapten, bergaptol has the least potency to inhibit cytochrome P450 CYP3A4 [60]. Instead, bergaptol inhibited drug efflux transporters, such as P-glycoprotein (P-gp) or MRP2 function, resulting in the increased uptake of vinblastine [60,62]. Previous studies showed significant overlap in the substrate and inhibitor specificities of CYP3A and P-gp [63]. The specific inhibitory effect of 0 to 20 μM of bergaptol on P-gp with minimal effect on CYP3A4 activity is intriguing. The effect of a combination between bergaptol and anti-cancer drugs that use P-gp-mediated efflux warrants further investigation for its potential to overcome drug resistance.

### 3.4. Anti-Osteoporosis Effects

Osteoporosis is a metabolic bone disease that results from a decrease in bone density and mass or deterioration of the structure of bone tissue [64]. An in vitro study using CCK8 assays, TRAP staining experiments, and ALP activity assays showed that bergaptol can suppress osteoclast formation and stimulate osteoblastic differentiation [64]. Since these are biomarkers of bone formation [65], such findings suggest that bergaptol may have the potential to prevent and treat osteoporosis. According to KEGG pathway enrichment analysis, the PI3K-Akt signaling pathway was identified as the most important pathway in these results. Simultaneous activation of the PI3K/AKT signaling pathway can increase the content of osteogenic alkaline phosphatase (ALP), enhance mineral formation, and upregulate the expression of bone-formation-related genes [64]. Consistent with the findings in primary osteoblastic cell studies, bergaptol can increase ALP activity, type 1 collagen synthesis, bone nodule formation, and bone morphogenetic protein-2 (*BMP-2*) gene expression in a dose- and time-dependent manner [15,66]. Interestingly, a combination of active ingredients of bergaptol and other compounds including osthole, columbianadin, notopterol, isoimperatorin, psoralen, xanthotoxin, bergapten, and nodakenin was shown to reduce bone resorption, bone dissolution, and alterations in bone surface and structure in a rheumatoid arthritis rat model [67]. Such positive effects are promising, so the clinical efficacy of bergaptol on bone health deserves further study.

### 3.5. Antilipidemic Effect

**Figure 5:**
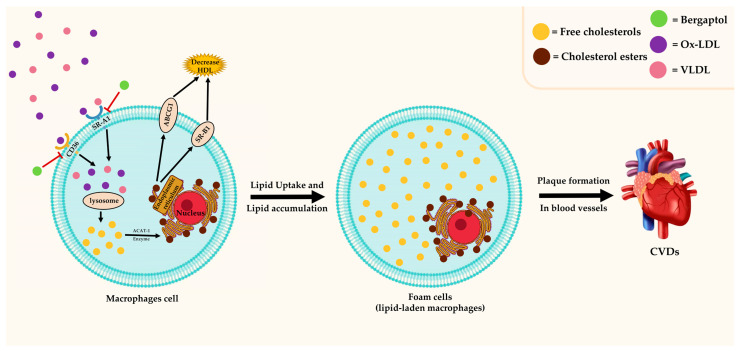
The role of bergaptol in foam cell formation. 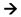 indicates activation, 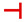 indicates inhibition.

Figure 5 summarizes the antilipidemic effect of bergaptol, which involves the inhibition of foam cell transformation from macrophages. Foam cells (lipid-laden macrophages) contain cholesterol and form a plaque that can lead to atherosclerosis [68]. Previous research has indicated that bergaptol can inhibit foam cell formation in RAW264.7 murine macrophage cells [31]. This effect is attributed to the attenuated cholesterol uptake by modulating scavenger receptor class A type I (SRA1) and cluster of differentiation 36 (CD36) [31]. Consistently, in vivo, studies of dyslipidemia Wistar rat models reported the antihyperlipidemic properties of bergaptol-containing plant extracts (*Exocarpium citri Grandis*, *Ficus religiosa*, and fermented lemon peel) [69,70,71,72]. The antilipidemic effect involves modulation of the inflammatory response, such as the TNF-α signaling pathway, which directly alters lipid metabolism [71,72,73]. Bergaptol-containing plant extract inhibits lipid uptake and accumulation as well as lipogenesis and stimulates lipolysis [71,72,73]. It is worth noting that the promising results of these extracts are probably derived from the combinatorial effects of multiple bioactive compounds (not just the bergaptol). More studies are warranted to investigate the actual effect of bergaptol and its clinical applications for dyslipidemia.

### 3.6. Antimicrobial

**Figure 6:**
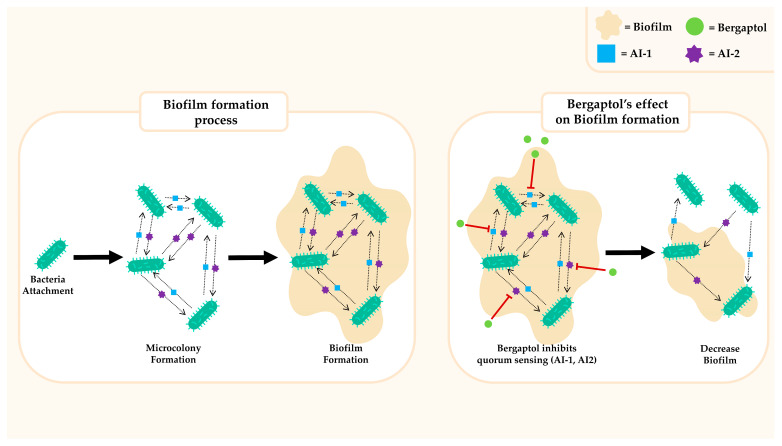
The role of bergaptol in inhibiting biofilm formation of bacteria. 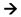 indicates activation, 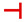 indicates inhibition.

Antibacterial activity is another important effect of furocoumarin [74]. Figure 6 summarizes the antibacterial activity of bergaptol, which involves the inhibition of quorum sensing and biofilm formation. A previous study reported that bergaptol can inhibit two auto-inducer systems [75]. Autoinduction (AI) or quorum sensing is the communication between cells and bacteria, which enhances the capabilities of pathogenic bacteria in combat against antibiotics and eukaryotic host defense mechanisms [76]. N-acyl-homoserine lactone (AI-1) and a furanosyl borate diester molecule (AI-2) are important autoinducer molecules that control gene expression for bioluminescence in *Vibrio harveyi* [77]. Based on a bioluminescence assay, bergaptol was found to inhibit AI-1 and AI-2 activity at 99.15 and 98.85%, respectively [75]. Previous reports suggest that AI molecules play an important role in biofilm formation [78]. They are commonly associated with various infections, especially in the urinary tract, catheters, dental plaque, and gingivitis [79]. The results of AI inhibition are consistent with the findings that bergaptol can inhibit biofilm formation. A previous study showed that among various types of furocoumarins, bergaptol had the highest capability to inhibit biofilm formation of *Pseudomonas aeruginosa* (at 32.65%) [75]. Furan forms are important structures of furocoumarin that interfere with the autoinduction systems of bacteria. Their structure resembles autoinducer molecules (AI-1 and AI-2), resulting in competitive binding which in turn could lead to the inhibition of quorum sensing [80,81]. This unique property of bergaptol warrants further studies for its application in infection control.

### 3.7. Neuroprotective Effect

Chronic neuroinflammation especially in the hippocampal region is a key mechanism of various neurological and cognitive impairments [82,83]. Microglia, as innate immune cells, play a crucial role in mediating neuroinflammation, but the overstimulation of microglia can also lead to neurological diseases [35,84]. A previous study reported that bergaptol can reduce the density of Iba-1-positive microglia in the hippocampal region of LPS-treated rats. This reduction in microglial density results in decreased levels of inflammatory factors such as TNF-α, IL-6, and IL-1β, leading to the inhibition of neuronal death and synaptic dysfunction [35]. Furthermore, recent research has suggested that atherosclerosis is established as a major risk factor for neurological diseases [85]. Therefore, the ability of bergaptol to inhibit foam cell formation may represent another potential neuroprotective role deserving further investigation. Nevertheless, it is worth noting that bergaptol was unable to protect neurons against amyloid β-mediated neurotoxicity [86]. Thus, its potential application may be more relevant to neurological diseases with inflammation as an underlying mechanism.

## 4. Toxicity

In general, furocoumarins are known for their phototoxic (transforming into cytotoxic products causing cell death when activated by solar light) and photomutagenic (causing gene mutation when irradiated by visible or UV light) properties [87]. Consistently, two prospective cohort studies conducted in the USA (involving a total of 122,744 participants) reported a significant association between high consumption of total furocoumarins and an increased risk of skin cancer (basal cell carcinoma) [88]. However, this study had several limitations, including the estimation of consumption based on the Food Frequency Questionnaire (FFQ), a self-reported measurement subject to potential misclassification. The FFQ is relatively subjective. Some respondents may not be able to distinguish similar products that contain different amounts of total furocoumarins. For example, regular orange juice contains 3.2 ng/g while fortified orange juice contains 2.4 ng/g of total furocoumarin. Lime juice contains 14,579 ng/g while lemon juice contains 1560.8 ng/g of total furocoumarin. Their confusion could lead to mistakes in the consumption data and consequently affect the results relating to furocoumarin exposure. Additionally, the FFQ is highly memory-intensive, making it prone to errors in both the quantity and frequency of consumption, especially for lengthy recall periods such as every 2–4 years like in this study [89]. It is noteworthy that only 10 furocoumarin-containing food items were identified in the FFQ in the study, potentially not covering all sources of furocoumarins consumed by the participants [88]. Furthermore, food sources of furocoumarins also contain other compounds. Thus, the increased cancer risk may be attributed to other substances or the combination effect with other compounds. Therefore, determining toxicity based on a few cohort studies may not be justified, and more studies are needed to clarify this issue.

In contrast to the general perception of furocoumarin, an in vitro study reported that bergaptol did not exhibit phototoxicity or photomutagenicity in V79 lung fibroblast cells within the tested concentration range (0–100 µg/mL) [90]. Furthermore, an in vivo study did not find any toxicities when bergaptol was given to mice at a concentration of 40 mg/kg (2-week IP injection) for 4 weeks [35]. To our knowledge, acute and chronic oral toxicity studies of bergaptol are still lacking. Owing to its potential health benefits and its unique properties different from other furocoumarins, future toxicity testing of bergaptol according to OECD guidelines is encouraged.

Like other furocoumarins, bergaptol has been reported to inhibit cytochrome P450 (CYP) specifically on CYP2C9 and CYP3A4 [90,91]. These enzymes are involved in the metabolism of a wide range of drugs [92,93]. Inhibition of CYP results in reduced metabolism of compounds that serve as substrates for this enzyme, potentially leading to elevated concentrations of drugs or toxicants in the body [92,93]. Such an effect can heighten the risk of adverse effects or toxicity associated with these substances [90,92]. The IC_50_ values of furocoumarins for inhibiting CYP are lower than those of other phytochemicals, e.g., flavonoids and limonoids, because of more non-polarity [94]. The IC_50_ values of bergaptol for CYP2C9 and CYP3A4 inhibition are within the ranges of 9.92 to 50.00 µM and 24.92 to 77.50 µM, respectively [37,90,91,94]. According to clinical evidence, the interaction between drugs and grapefruit juice (containing bergaptol) occurs when a drug is significantly first-pass metabolized by enteric CYP3A enzymes [95]. The inhibition of CYP3A4 by furanocoumarins demonstrates a competitive pattern by binding between the active sites of the enzyme and the olefin region, 6′,7′-position,2,3-furan moiety, and lactone ring [96]. Bergaptol can induce NADPH-dependent irreversible inhibition of CYP2C9 by covalent modification with furanoepoxide 2 and γ-ketoenal 3 [91]. Nonetheless, the impact of bergaptol on CYP enzymes varies with factors such as time, concentration, route of administration, biological factors, and individual differences [91,97].

Based on the above-mentioned evidence, bergaptol may be capable of enhancing or prolonging the bioavailability of certain drugs. Thus, it may have clinical application as a sensitizer or adjuvant to some treatments such as cancer chemotherapy. It may help lower the effective doses and adverse effects of those drugs. Paradoxically, consuming bergaptol-containing food together with CYP2C9 and CYP3A4-metabolized drugs may potentiate toxicity of the drugs and pose health risks. To safely and effectively use bergaptol for health benefits, more studies are required to explore the toxicity of bergaptol as well as its interactions with other drugs.

## 5. Methodology

This review collected the scientific literature published before September 2023 on the pharmacology and toxicity of bergaptol. No period limitation was considered in this investigation. All relevant information on bergaptol was gathered from widely accepted scientific search engines and databases. Most of the cited information in this article was from peer-reviewed published journals. We did not limit this review to studies of pure bergaptol; it also includes in vitro, in vivo, and clinical studies of bergaptol-containing plants, plant extract, oil, and juice.

## 6. Conclusions and Future Perspectives

Bergaptol, a natural furocoumarin predominantly found in citrus plants such as grapefruit, bergamot, lemon, and lime, exhibits diverse biological properties with potential therapeutic applications. Numerous in vitro studies have explored its efficacy and possible mechanisms of action. Bergaptol inhibits inflammatory responses by suppressing expression of *COX-2* and *iNOS* genes and reducing cytokine formation via inhibiting JAK2, STAT3, and p65 pathways. Bergaptol reduces several types of ROS and exhibits antioxidant properties through the HAT and SPLET mechanisms, contingent on the polarity of the medium. Bergaptol has anti-cancer activity by induction of G1/S phase cell cycle arrest and apoptosis via regulating the expression of relevant genes and proteins (such as *BCL2*/*Bax*, *caspase-3*, *caspase-9*, and *Skp2*). Interestingly, the ability of bergaptol to inhibit P-gp-mediated efflux suggests its potential to overcome resistance to chemotherapy. Bergaptol also exhibits antiosteoporotic properties by promoting osteoblastic differentiation via PI3K/AKT-mediated upregulation of bone-formation-related genes. Bergaptol has antilipidemic properties by altering lipid metabolism, particularly cholesterol, inhibiting foam cell formation, and modulating inflammatory response. Interestingly, among many types of furocoumarins, bergaptol has the highest potential for inhibition of quorum sensing. This property makes it an interesting candidate for antimicrobial agents. More in vivo and clinical studies are needed to explore its application in quorum-sensing-mediated infectious diseases such as those caused by *Pseudomonas aeruginosa* and *Staphylococcus aureus* [95]. Owing to its effect in controlling neuroinflammation, bergaptol may have potential for the prevention of Alzheimer’s disease, Parkinson’s disease, and amyotrophic lateral sclerosis [95]. Though compelling evidence suggests the potential health benefits of bergaptol, the toxicity of bergaptol is scarcely known. A few studies have addressed the inhibitory activity of bergaptol against CYP enzymes. There are neither published subchronic nor chronic animal toxicity tests nor clinical safety studies of bergaptol.

By structure, bergaptol resembles psoralen and bergapten since it can be synthesized from those compounds [3,4,5,6,7]. Therefore, after consumption into the body and passing biotransformation in the liver, bergaptol metabolites should be relatively similar to those of psoralen and bergapten. Interestingly, an in silico study has predicted that metabolites of psoralen and bergapten include furan, resorcinol, phenyl ester, epoxide, and coumarin [98]. If continuously consumed, these metabolites could induce carcinogenicity, skin sensitization, hepatotoxicity, and photoallergenicity. Though existing in vitro studies did not find any potential toxicities, adverse effects could still be expected when bergaptol is consumed by any living organism. Thus, further in vivo toxicity testing and clinical safety trials are needed with special attention paid to its potentially harmful metabolites.

Based on the existing literature, further studies at the in vivo and clinical levels are necessary to confirm and elucidate the potential of bergaptol. Comprehensive research is crucial for understanding potential side effects, determining optimal dosages, and evaluating long-term safety, particularly when considering its application in therapeutics. This holds importance, especially for substances affecting CYP activity (including bergaptol), which is a key factor in causing pharmacokinetic drug–drug interactions. The continuous research effort will significantly contribute to unlocking the complete pharmacological potential of bergaptol and ensuring its safe and effective utilization in future applications.
